# Minnesota Dental Law

**Published:** 1885-08

**Authors:** 


### Minnesota Dental Law.

The following is the text of an "Act to insure the better education of practitioners of dental surgery and to regulate the practice of dentistry in the State of Minnesota:"

*Be it enacted by the Legislature of the State of Minnesota:*

SECTION 1. That it shall be unlawful for any person who is not at the time of the passage of this act engaged in the practice of dentistry in this State to commence such practice unless he or she shall have obtained a certificate as hereinafter provided.

SEC. 2. A board of examiners, to consist of five practicing dentists, is hereby created, whose duty it shall be to carry out the purposes and enforce the provisions of this act.

The members of said board shall be appointed by the Governor, who shall select them from ten candidates whose names shall be furnished him by the State Dental Association; at least three members of said board shall be members of the State Dental Association. The term for which the members of said board shall hold their offices shall be five years, except that the members of the board first to be appointed under this act shall hold their offices for the term of one, two, three, four, and five years respectively, and until their successors shall be duly appointed. In case of a vacancy occurring in said board, such vacancy shall be filled by the Governor from names presented to him by the

Minnesota State Dental Association. It shall be the duty of the Minnesota State Dental Society to present twice the number of names to the Governor of those to be appointed.

SEC. Said board shall chose one of its members president and one the secretary thereof; and it shall meet at least once in each year, and as much oftener and at such times and places as it may deem necessary. A majority of said board shall at all times constitute a quorum, and the proceedings thereof shall at all reasonable times be open to public inspection.

SEC. 4. Within six months from the time that this act takes effect, it shall be the duty of every person who is at that time engaged in the practice of dentistry in this State to cause his or or her name and residence or place of business to be registered with said board of examiners, who shall keep a book for that purpose.

The statement of every such person shall be verified under oath before a notary public or justice of the peace in such manner as may be prescribed by the board of examiners. Every person who shall so register with said board as a practitioner of dentistry may continue to practice the same as such without incurring any of the liabilities or penalties provided in this act, and shall pay to the board of examiners for such registration a fee of one dollar. It shall be the duty of the board of examiners to forward to the clerk of the court of each county in the State a certified list of the names of all persons residing in this county who have registered in accordance with the provisions of his act, and it shall be the duty of all clerks to register such names in a book to be kept for that purpose.

SEC. 5. Any and all persons who shall so desire may appear before said board, at any of its regular meetings, and be examined with reference to their knowledge and skill in dental surgery; and if the examination of any such person or persons shall prove satisfactory to said board, the board of examiners shall issue to such persons as they shall find to possess the requisite qualifications a certificate to that effect, in accordance with the provisions of this act. Said board shall also indorse as satisfactory diplomas from any reputable dental college, when satisfied with the character of such institution, upon the holder of such diploma furnishing evidence satisfactory to the board of his or her right to the same.

All certificates issued by said board shall be signed by its officers, and such certificate shall be prima facie evidence of the right of the holder to practice dentistry in the State of Minnesota.

SEC. 6. Any person who shall violate any of the provisons of this act shall be deamed guilty of a misdemeanor, and upon conviction may be fined not less than fifty dollars or more than two hundred dollars, or be confined six months in the county jail. All fines received under this act shall be paid into the common school fund of the county in which such conviction takes place.

SEC. 7. In order to provide the means for carrying out and maintaining the provisions of this act, the said board of examiners may charge each person applying to or appearing before them for examination for a certificate of qualification a fee of ten dollars, which fee shall in no case be returned; and out of the funds coming into the possession of the board from the fees so charged the members of said board may receive as compensation the sum of five dollars for each day actually engaged in the duties of their office, and all legitimate and necessary expenses incurred in attending the meetings of said board. Said expenses shall be paid from the fees and penalties received by the board under the provisions of this act, and no part of the salary or other expenses of the board shall ever be paid out of the State treasury. All moneys received in excess of said per diem allowance and other expenses above provided for shall be held by the secretary of said board as a special fund for meeting the expenses of said board and carrying out the provisions of this act, he giving such bond as the board shall from time to time direct. And said board shall make an annual report of its proceedings to the Governor by the 15th of December of each year, together with an account of all moneys received and disbursed by them pursuant to this act.

SEC. 8. Any person who shall receive a certificate of qualification from said board shall cause his or her certificate to be registered with the clerk of the court of any county or counties in which such persons may desire to engage in the practice of dentistry, and the clerks of the court of the several counties in his State shall charge for registering such certificate a fee of twenty-five cents for such registration. Any failure, neglect, or refusal on the part of any person holding such certificate to register the same with the clerk of the court, as above directed, for a period of six months, shall work a forfeiture of the certificate, and no certificate when once forfeited shall be restored, except upon the payment to the said board of examiners of the sum of twenty-five dollars as a penalty for such neglect, failure, or refusal.

SEC. 9. Any person who shall knowingly and falsely claim or pretend to have or hold a certificate of license, diploma, or degree granted by any society, or who shall falsely and with intent to deceive the public claim or pretend to be a graduate from any incorporated dental college, not being such graduate, shall be deemed guilty of a misdemeanor and shall be liable to the same penalty as provided in section six of this.

SEC. 10. This act shall take effect and be in force from and after its passage.

Approved March 3, 1885.

The Governor has appointed the following Board of Examiners: J. L. Clements, D.D.S., President, Fairbault; J. H. Martindale, M.D., D.D.S., Secretary, Minneapolis; G. V. I. Brown, D.D.S., St. Paul; B. G. Merry, D.D.S., Stillwater; M. R. Metcalf, D.D.S., Duluth. The board will be in session, at Minneapolis, July 31 and August 1 and 3, 1885, for the purpose of issuing licenses to those holding diplomas, and to examine such as may apply, in accordance with the provisions of the law.

